# Preparation of Nano-SiO_2_/Al_2_O_3_/ZnO-Blended PVDF Cation-Exchange Membranes with Improved Membrane Permselectivity and Oxidation Stability

**DOI:** 10.3390/ma11122465

**Published:** 2018-12-04

**Authors:** Xuemin Zhang, Jian Zhou, Xin Zou, Zhongyu Wang, Yunchen Chu, Sanfan Wang

**Affiliations:** 1 Engineering Research Center of Water Resources Utilization in Cold and Drought Region, Ministry of Education, School of Environmental and Municipal Engineering, Lanzhou Jiaotong University, No. 88, Anning West Road, Lanzhou 730070, China; luck849200@126.com (J.Z.); 18219710232@163.com (X.Z.); chuyunchen95@163.com (Y.C.); sfwang1612@163.com (S.W.); 2 Urban and Rural Planning Bureau of Mudanjiang, No. 41, Wusuli Road, Mudanjiang 157000, China; izhongyu@163.com

**Keywords:** poly (vinylidene fluoride), cation-exchange membrane, nano-SiO_2_/Al_2_O_3_/ZnO, membrane performance

## Abstract

Ion exchange membranes are used in practically every industry; however, most of them have defects such as low permeability and poor oxidation resistance. In this paper, cation-exchange membranes were prepared with poly (vinylidene fluoride) (PVDF) blended with nano-SiO_2_, nano-Al_2_O_3_ and nano-ZnO. Sulfonic acid groups were injected into the membrane prepared by styrene grafting and sulfonation. The methods used for characterizing the prepared membranes were Fourier transform infrared spectroscopy (FTIR), scanning electron microscopy (SEM), and electrochemical measurements. Membrane performance, such as the ion exchange capacity (IEC), water uptake (WU), transport number, membrane permselectivity, membrane resistance, functional groups, and morphology were also evaluated. The hydrophilia, IEC, and permselectivity of cation-exchange membranes depended on the nanoparticle content of the membrane matrix. High transport property values were obtained, which increased with increasing nano-SiO_2_/Al_2_O_3_/ZnO weight fractions. Finally, the cation-exchange membranes prepared with 1.5% nano-SiO_2_, 2.0% nano-Al_2_O_3_ or 2.0% nano-ZnO all exhibited excellent membrane properties, including membrane permselectivity (PVDF/2% ZnO-g-PSSA membranes, 94.9%), IEC (PVDF/2% Al_2_O_3_-g-PSSA membranes, 2.735 mmol·g^−1^), and oxidation resistance (PVDF/1.5% SiO_2_-g-PSSA membranes, 2.33%). They can be used to separate applications in a variety of different areas, such as water treatment, electro-driven separation, heavy metal smelting, or other electrochemical processes.

## 1. Introduction

Separation processes based on membranes are used in the most advanced industry processes, such as wastewater treatment and membrane electrode position processes [1,2,3]. Ion exchange membranes (IEMs) are modern separation membranes and have been used since the last century in many industrial fields for various separations, such as ions from non-ionic substances and acidic gases like carbon dioxide, by carrier transport [4,5]. The incorporation of nanomaterials in the preparation of ion exchange membranes to improve membrane performance has been widely discussed. Hosseini et al. [6] prepared PVC/TiO_2_ nanoparticle mixed matrix cation exchange membranes to improve membrane mechanical strength. Zuo et al. [7] adopted the blending method to prepare poly (vinylidene fluoride) (PVDF)-SiO_2_ hybrid anion-exchange membranes, which exhibited better water content, ion-exchange capacity, conductivity, and mechanical properties. Cseri et al. [8] used a graphene oxide (GO) and polybenzimidazolium nanocomposite to prepare anion exchange membranes (AEMs) that were mechanically robust and highly permselective.

The typical cation-exchange groups are sulfonic acids (–SO_3_H), carboxylic acids (–COOH), phosphonic acids (–PO_3_H_2_), and phenolic hydroxide groups. –SO_3_H was selected as the cation-exchange group in this paper. In general, cation IEMs should have the following properties: high permselectivity, good chemical stability, low electrical resistance, and good form and mechanical stability. Nafion membranes with high conductivity and selectivity are expensive [9,10,11,12]. Therefore, for practical applications and academic research, the development of low cost and high-selectivity cation exchange membranes with simple preparation methods is essential.

In general, polymers such as PVDF have excellent properties such as a high thermal stability, good chemical resistance, radiation resistance, good membrane formation, and low cost, and are widely used as a base material for the preparation of IEMs [13,14,15]. However, the hydrophobicity of PVDF is a growing problem: the appetency of organics for PVDF membranes leads to increased IEM resistance and membrane fouling, and increases the energy consumption of an IEM used in certain industries (such as the metallurgical industry) [16,17]. There are many methods that can be used for the modification of PVDF membranes, such as irradiation [18]. Currently, there are numerous reports on PVDF membrane modification by mixing the polymer with inorganic materials [7,19,20]. Organic nanoparticles are widely used because of their excellent properties, including good mechanical properties and stable chemical properties [21]. Moreover, organic and inorganic materials can be used to prepare homogeneous IEMs with high permeability, good oxidation resistance, high hydrophilicity, small membrane resistance, and good surface morphology. The inorganic nanomaterials that can be mixed with PVDF are numerous, and include nano-SiO_2_. However, little research has investigated the modification of a PVDF cation-exchange membrane by blending different amounts and types of inorganic nanoparticles [20,22,23].

In this work, cation-exchange membranes based on PVDF were prepared with different contents of nano-SiO_2_/Al_2_O_3_/ZnO. Due to the inherent properties of the two components, these nano-SiO_2_/Al_2_O_3_/ZnO/PVDF membranes were expected to have excellent physicochemical and electrochemical properties [24]. The influence of the SiO_2_, Al_2_O_3_ and ZnO weight fractions on the properties of these membranes (i.e., selective permeability, antioxidant properties, ion exchange capacity (IEC), membrane conductivity, and water uptake (WU) was extensively studied.

## 2. Experimental Method

### 2.1. Materials and Reagents

PVDF (Mw = 1,000,000) was bought from Arkema, Paris, France. The nano-SiO_2_/Al_2_O_3_/ZnO (99.9%, 30 nm) was used as received (Xiya Reagent, Linyi, Shandong China). Analytical grade N,N-dimethylformamide (DMF) and benzoyl peroxide (BPO) were bought from Guanghua Reagent (Guangzhou, China). Analytical grade styrene and tetrahydrofuran were purchased from Shanghai Zhongqin Chemical Reagent (Shanghai, China). Distilled water was used from beginning to end. All other reagents were of analytical grade.

### 2.2. Preparation of the Membranes

A specified amount of nano-SiO_2_/Al_2_O_3_/ZnO was dispersed in 80 mL of DMF by an ultrasonicator and intermittently stirred. Then, 20 g of PVDF was dissolved into it, and after degassing, a uniform casting solution was obtained. The casting solution was poured on a glass plate to obtain a film with the desired thickness, which could be achieved by pre-setting the membrane scraping machine, and the films and glass plates were placed in an oven at 60 °C for 90 min. The PVDF-based membranes were removed from the glass plates by immersing the plates in water. The membranes were soaked in a 0.1 mol·L^−1^ ethanol solution of NaOH at 70 °C for 75 min to form the C–C double bonds. Then, styrene was grafted onto the membranes using a mixed solution of BPO, 400 mL of styrene, and 100 mL of tetrahydrofuran at 70 °C for 14 h. The grafting rate was 34%, which was calculated by the gravimetric method [25]. Finally, the PVDF-g-PS membranes were soaked in 98% H_2_SO_4_ at 70 °C for 8 h to obtain cation-exchange membranes containing sulfonic acid groups. The sulfonation rate was 98%, which was calculated by the method of acid-base titration [25]. The PVDF-g-PSSA membranes were stored in deionized water for 24 h for further characterization.

**Figure 1:**
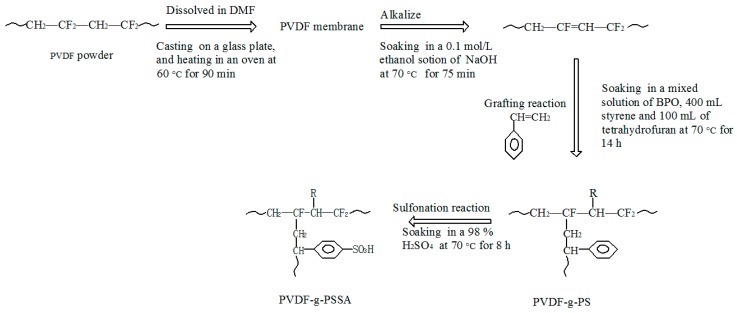
The membrane preparation principle.

The membranes were designated PVDF/X% SiO_2_/Al_2_O_3_/ZnO-g-PSSA, where X is the SiO_2_/Al_2_O_3_/ZnO content (%) of the PVDF in the membrane. PVDF-g-PSSA represents a sulfonated membrane without nanoparticles. The membrane preparation principle is shown in Figure 1.

### 2.3. Membrane Characterization

#### 2.3.1. Transport Number and Membrane Permselectivity

The permselectivity was characterized by the number of migrations [26]. The number of migrations (the cations) is the percentage of ions moving through the membrane, which can be used to characterize the selectivity of the membrane for ions with a heterogeneous charge. The number of cations is calculated by measuring the membrane potentials. The membrane was soaked in 0.15 M NaCl until it became a K^+^-type membrane (the main exchange group of the membrane is K^+^). The prepared membrane was fixed between two compartments of an electrolyzer. The effective part of the prepared membrane was 1 cm^2^. An electrolyte solution with a concentration of C_1_ = 0.1 mol·L^−1^ and a solution of C_2_ = 0.01 mol·L^−1^ were poured into the compartments on both sides of the membrane at 25 °C. The two compartments were separately agitated using a magnetic stirrer to minimize the effect of the boundary layer on the membrane surface. A saturated calomel electrode was placed in each of the two compartments, and the membrane potential generated by the difference in concentration between the two compartments was measured using a digital automatic multimeter (model: UNI-TUT33D, Digital Multimeter, Chendu, China). The measurement was taken every 5 min, and the potential was recorded every minute to obtain a changeless value [27,28]. Finally, the number of cations moving through the membrane can be obtained from Equation (1):

(1) $$t^{+}=\frac{E_{m}-E_{0}}{2E_{0}}$$

where *E_m_* is the membrane potential between the two compartments and *E_0_* is the ideal membrane potential between the electrodes.

*E_0_* can be obtained from Equation (2):

(2) $$E_{0}=\frac{\mathrm{RT}}{\mathrm{nF}}\left(2t-1\right)\mathrm{In}\frac{\alpha_{1}}{\alpha_{2}}$$

where R is the universal gas constant (~8.314 J·K^−1^·mol^−1^), T is the Kelvin temperature, F is the Faraday constant (~96,485.3 C·mol^−1^), n is the electrovalence of counterion, $\alpha_{1}$ and $\alpha_{2}$ are the activities of the electrolyte, and *t^+^* is the number of cations moving through the membrane [29].

The ion selectivity of the membrane can be quantified as the selectivity of the counterion through the IEM, as found by Equation (3) [30,31]:

(3) $$P=\frac{t^{+}-t}{1-t}$$

where *t* is the solution transport number. At 25 °C, the solution transport number of NaCl is 0.39 for its cation in water [32].

#### 2.3.2. IEC and WU 

The IEC, which represents the quantity of milliequivalents for fixed charges in 1 g of the dry membrane, was decided by the most common method—acid base back titration under the action of an indicator (phenolphthalein) [33]. In a typical procedure, the studied membranes were equilibrated in 1.0 mol·L^−1^ HCl or 1.0 mol·L^−1^ NaOH solutions to change the membrane into its H^+^ or OH^−^ type. The membranes were rinsed with deionized water (about 10 L·m^−2^) to dislodge needless HCl or NaOH, and then soaked in distilled water until all acids or bases were removed. Then, the membranes were balanced in 0.1 M NaCl for 24 h. The increase of acidity or basicity of the membrane decided the IEC. The IEC of the membranes was calculated using Equation (4):

(4) $$IEC=\frac{C_{NaOH}\times V_{NaOH}}{m_{dry}}$$

where *C* and *V* are the concentration and volume of the NaOH used on the membranes, respectively, and *m* is the mass of the dried membrane.

The membranes were immersed in distilled water for 24 h or more. Then, after the distilled water on the membrane surface was absorbed with filter paper, the wet membrane was weighed. The wet membranes were placed in an oven at 60 °C until the weight of the membrane was constant. The WU of the membranes was calculated using Equation (5):

(5) $$WU=\frac{W_{wet}-W_{dry}}{W_{dry}}$$

where *W_wet_* is the wet weight of the membrane and *W_dry_* is the dry weight of the membrane.

#### 2.3.3. Membrane Area Resistance

The membrane resistance is an important parameter for evaluating membrane performance, and it is important in the practical applications of IEMs [34,35]. The membrane resistance was calculated using an electrochemical workstation (PGSTAT128N, Metrohm, Beijing, China). The membrane resistance measurements were conducted by an electrolytic cell. The electrolytic cell system consisted of two compartments with dimensional stability electrodes (DSEs). The membranes were used to divide the cell into two compartments. The electrolyte solution was a 2 M NaCl solution. Before the experiments, the studied membranes were stored in distilled water for 24 h or more and soaked in the electrolyte solution for at least 24 h to equilibrate. The resistances of the electrolyte solution and the membranes in the electrolyte solution were measured. 

The area resistance of the membrane (Ω·cm^2^) was determined using Equation (6):

(6) $$R_{m}=\left[\frac{R_{1}R^{\prime }}{R^{\prime }-R_{1}}-\frac{R_{0}R^{\prime }}{R^{\prime }-R_{0}}\right]\times \frac{\pi D^{2}}{4}$$

where *R_m_* is the membrane area resistance, *R_1_* is the measured resistance of both the membrane and the electrolyte solution, *R_0_* is the measured resistance of the electrolyte solution, *R’* is a variable resistance that does not change during the measurement process, and *D* is the cross-sectional diameter of the cell (cm^2^).

#### 2.3.4. Scanning Electron Microscopy (SEM)

The surface morphology and structure of the thoroughly dried membranes were observed using a scanning electron microscope (Zeiss Ultra Plus, Carl Zeiss, Jena, Germany) after the sputter deposition of a thin, conductive gold coating on the membranes. The samples were vacuum-dried prior to the tests.

#### 2.3.5. Fourier Transform Infrared Spectroscopy (FTIR)

The FTIR spectra of the different membranes were obtained using an FTIR spectrometer (IRPrestige-21, SHIMADZU, Tianjin, China) with the arc reflection technique. The membranes were dried in an oven before testing. The spectra were analyzed to study the changes in the groups or chemical bond structures.

#### 2.3.6. Membrane Oxidative Stability and Burst Strength

To estimate the stability of the prepared membranes, they were immersed in a 3% H_2_O_2_ aqueous solution with 4 ppm Fe^3+^ at 25 °C for 60 h, then dried at 80 °C for 4 h. The constant weights of the dried membrane before and after the modification were obtained (Model: BSM-220.4, Mettler Toledo Group, Zurich, Switzerland). The weight decrease percentage indicates the membrane oxidative stability [33,36]. The burst strength of the membranes was determined by a bursting strength tester (Hengke, Dongguan, China).

## 3. Results and Discussion

### 3.1. Transport Number and Membrane Permselectivity

**Figure 2:**
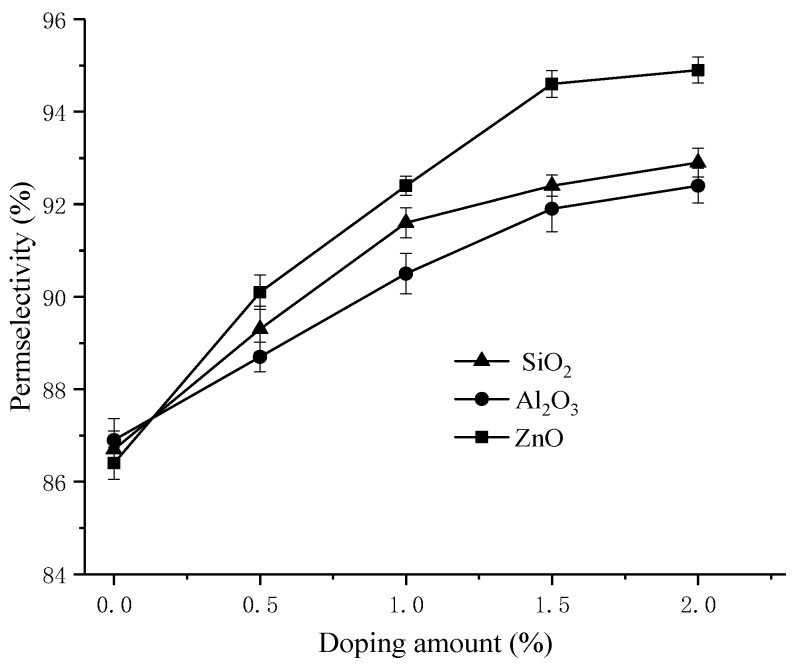
The effect on membrane permselectivity under different doping amounts of nano-SiO_2_, nano-Al_2_O_3_ and nano-ZnO.

The “membrane potential excluded-pore closed” theory, proposed by Wang [37], indicates that the membrane permselectivity can be improved by decreasing the membrane pore size to prevent ion leakage, which can be caused by oversized pores. The transport number and permselectivity of membranes are shown in Table 1 and Figure 2.

**Figure 3:**
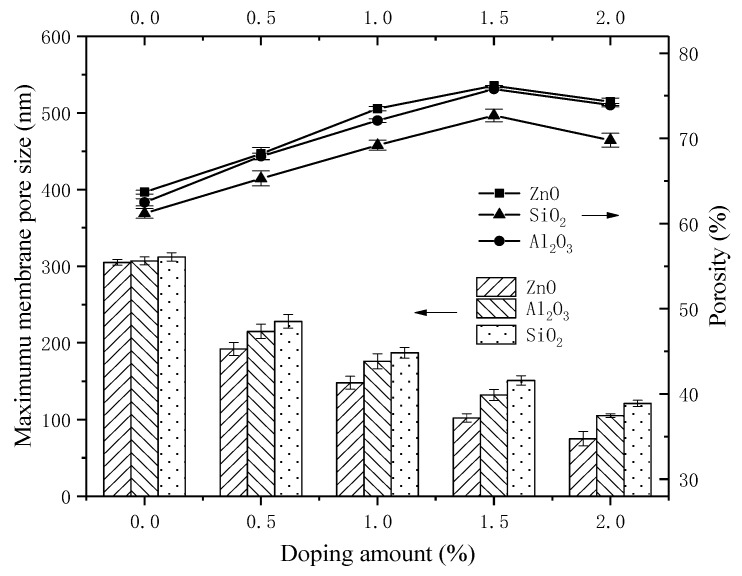
The effect of maximum pore size and porosity of prepared membranes under different doping amounts of nano-SiO_2_, nano-Al_2_O_3_ and nano-ZnO.

As shown in Table 1 and Figure 2, the membrane potential, the transport number, and permselectivity increased with an increase in nanoparticle content. The increasing tendency slowed at a doping amount of 2.0%. This finding may be interpreted as reflecting the membrane pore size. Blending IEMs with inorganic nanoparticles can reduce the maximum membrane pore size and prevent leakage due to excessive membrane pore size. Moreover, the porosity increased with the weight fraction of nanoparticles, which can also be seen in Figure 3, and may also influence the improvement of membrane permselectivity.

The results showed that the influence of the membrane pore size is remarkable in our prepared membranes, resulting in an increase in the transport number and permselectivity of the counterions. This is consistent with the results found by Zuo et al. [29] in the preparation of organic-inorganic membranes. The PVDF organic-inorganic membranes exhibited better transport properties, which may be useful in electro-driven separation or for other electrochemical processes [38]. In contrast, the PVDF/2% ZnO-g-PSSA membrane in this paper has a better permselectivity of 0.949, increased by 9.8% compared to PVDF-g-PSSA membranes. This may be due to the fact that nano-zinc oxide is more easily combined with PVDF.

Membrane permselectivity is related to the pore size and porosity of the membranes [30]. The membrane pore size was determined by the bubble method; the device was described in the patent [39]. The maximum membrane pore size was calculated by the Laplace formula, as in Equation (7):

(7) $$R_{Max}=\frac{2\sigma COS\theta }{P}$$

where *R_Max_* is the maximum membrane pore size, σ is the interfacial tension, *θ* is the contact angle, and *P* is the pressure when the first bubble appeared.

The porosity was calculated by the Laplace formula, as in Equation (8):

(8) $$\rho =\frac{m_{1}-m_{2}}{A\delta \rho H_{2}O}\times 100\%$$

where ρ is the porosity, $m_{1}$ is the quality of the dry membrane, $m_{2}$ is the quality of the wet membrane, *A* is the membrane area, and *δ* is the membrane thickness.

The maximum membrane pore sizes and porosity of the different types of membranes are shown in Figure 3.

As seen in Figure 3, with the increase in the inorganic nanoparticle content in the membranes, the maximum membrane pore size decreased. In contrast, the difference in the maximum membrane pore size between mixed-nano-Al_2_O_3_ and mixed-nano-SiO_2_ is not significant, and the maximum membrane pore size of mixed-nano-ZnO is more obvious. This may be due to the fact that the chemical properties of nano-ZnO are comparably easier to combine with the membrane matrix [40]. At the same time, the porosity showed a trend of increasing initially and then decreasing. This may be attributed to the fact that inorganic nanoparticles have the function of a porogen, and a moderate doping amount can increase the porosity of the membrane [41]. In addition, the data were concentrated in Figure 2 and Figure 3. This indicates the small errors and good reproducibility of membranes.

The experimental results showed that a moderate reduction in maximum pore size and appropriate increase in porosity could improve the membrane permselectivity. Simultaneously, 2% inorganic nanoparticles in membranes, especially nano-ZnO, can significantly improve the membrane permselectivity.

### 3.2. IEC and WU Properties

**Figure 4:**
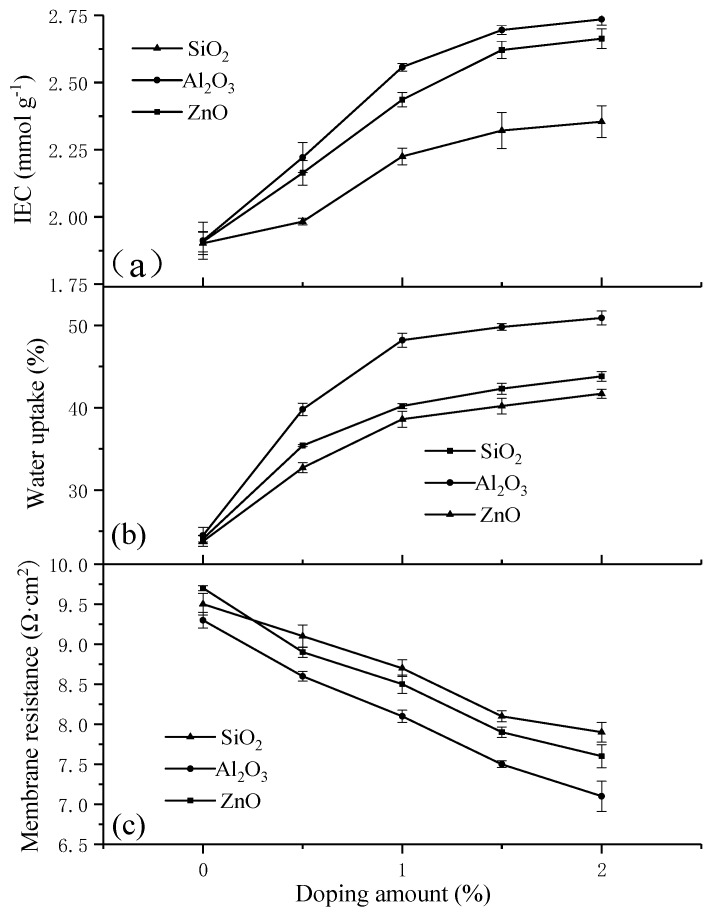
The effect on ion exchange capacity (IEC) (**a**), water uptake (WU) (**b**) and membrane resistance (**c**) of prepared membranes under different doping amounts of nano-SiO_2_, nano-Al_2_O_3_ and nano-ZnO.

IEM performance is interrelated to the IEC, because the number and the kind of ion exchange groups decide the membrane performance [42]. The IEC is an important indicator for evaluating the density of the active groups in the membrane, which are typical of the exchange ability of IEMs. To evaluate the IECs of different types of membranes, IEC experiments were performed. The thickness of the membranes was determined by the membrane scraping machine to be approximately 0.13 mm. The IEC values of all the prepared membranes are given in Figure 4a. 

As seen in Figure 4a, the IEC values improved with the increase in the nano-SiO_2_/Al_2_O_3_/ZnO content. This indicates that the physical and chemical nature of these membranes may be improved with an increase in inorganic nanoparticles. The IEC values ranged from 1.983 mmol·g^−1^ for PVDF/0.5% SiO_2_-g-PSSA to 2.735 mmol·g^−1^ for PVDF/2.0% Al_2_O_3_-g-PSSA. The IEC of PVDF/2.0% Al_2_O_3_-g-PSSA membranes increased by 43% compared to PVDF-g-PSSA membranes. Perhaps the chemical structure of nano-Al_2_O_3_ increases the charge density of the membranes, thereby improving the IEC [26]. Similar results were reported by Zuo et al.; that is, the IEC values improved with an increase in the nano-SiO_2_ [29]. 

For IEMs, WU is a significant index influencing the electrochemical performance of the membranes. The WU values of the membranes are listed in Figure 4b for the different types of membranes.

A high water content is known to increase the membrane conductivity and decrease the ion selectivity and the degree of crosslinking [26]. Figure 4b showed the change of water content for the different types of membranes. Compared to the PVDF-g-PSSA membranes, the WU of the membranes blended with inorganic nanoparticles obviously increased due to the increase in the number of hydrophilic sulfonic acid groups, because an increase in the inorganic nanoparticle content of the membranes leads to an obvious increase in the functional group density and the porous volume. Moreover, the increase of WU is also attributed to the hydrophilic group on the surface of the inorganic nanoparticles. This is consistent with the results found by Zhang et al. in the preparation of PVDF/ZnO hybrid membranes [22]. In addition, the IEMs blended with nano-Al_2_O_3_ had a more obvious increase than the other IEMs because nano-Al_2_O_3_ is an amphiphilic substance with strong hydrophilicity.

### 3.3. Membrane Area Resistance

Similar to the conductivity behavior of most commercial membranes, such as Nafion® (Puneng, Nanjing, China), the conductivity of our prepared membranes increased as the water content increased. Measuring the membrane conductivity is important in the assessment of the contributions of various functional groups [43]. The membrane area resistance data for the prepared membranes, equilibrated with a 2 M NaCl (25 °C) solution, are presented in Figure 4c.

As seen in Figure 4c, the membrane area resistance decreased as the nano-SiO_2_/Al_2_O_3_/ZnO content increased. This may be due to the increase in the SiO_2_/Al_2_O_3_/ZnO nanoparticles allowing exchange and conduction of ions in the membrane. According to related research, the conductivity of an IEM is affected by three factors: the network structure within the membrane, the relative size of the IEC, and the composition of the exchange group [26,44,45]. The strength of the electrostatic coactions between the functional group fixed on the membrane and the cations reportedly relies on the membrane surface charge density and the cation valence [46]. The membrane fixed-charge concentration increased with the SiO_2_/Al_2_O_3_/ZnO nanoparticle blending, and thus, the membrane area resistance decreased [26]. In addition, the decrease in the membrane resistance can also be attributed to an increase in the IEC and water content. The membrane blended with nano-Al_2_O_3_ had the lowest membrane resistance, which may be due to the high hydrophilicity of Al_2_O_3_ nanoparticles. Furthermore, the membrane area resistance of PVDF/2.0% Al_2_O_3_-g-PSSA membranes decreased by 22% compared to PVDF-g-PSSA membranes. As shown in Figure 4a–c, the data for IEC, WU and membrane resistance exhibit a small change which illustrates the low standard deviations and stable membrane properties.

### 3.4. SEM Studies

**Figure 5:**
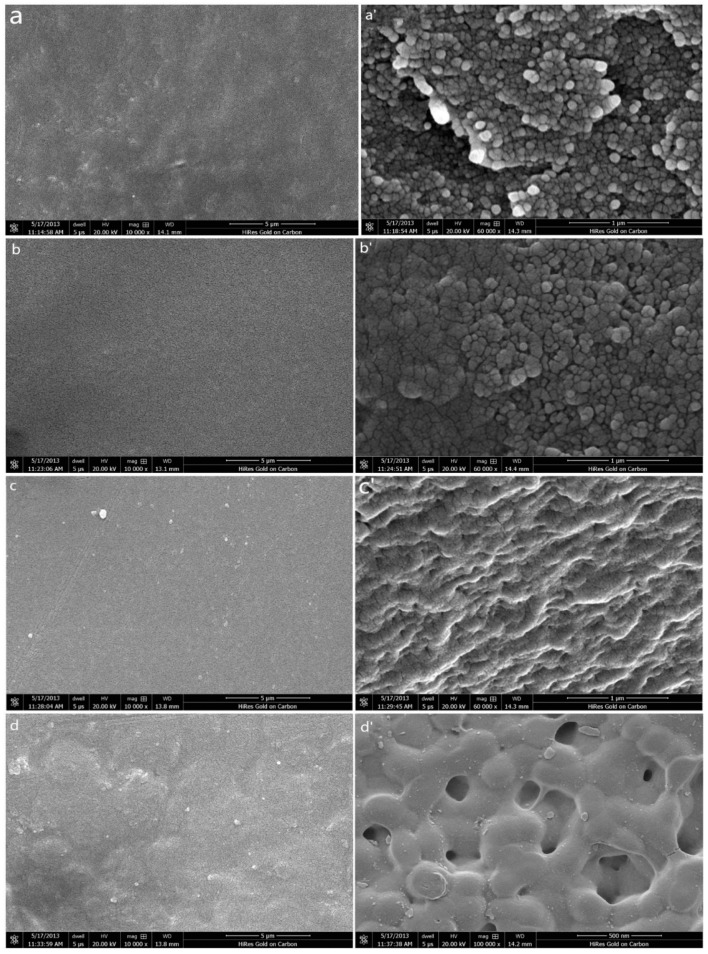
SEM images of prepared membranes (with the weight fraction 1% of non-organic nanoparticles taken as an example). (**a**) PVDF-g-PSSA (surface); (**a’**) PVDF-g-PSSA (cross-section); (**b**) PVDF/1.0% SiO_2_-g-PSSA (surface); (**b’**) PVDF/1.0% SiO_2_-g-PSSA (cross-section); (**c**) PVDF/1.0% Al_2_O_3_-g-PSSA (surface); (**c’**) PVDF/1.0% Al_2_O_3_-g-PSSA (cross-section); (**d**) PVDF/1.0% ZnO-g-PSSA (surface); (**d’**) PVDF/1.0% ZnO-g-PSSA (cross-section).

The SEM images of the surfaces and cross-sections of the prepared membranes are presented in Figure 5 (the mass fraction 1% of inorganic nanoparticles is taken as an example).

As Figure 5 shows, the effects of the nano-SiO_2_, nano-Al_2_O_3_ and nano-ZnO on the membrane morphology are explicitly seen in the SEM images. In the cross-sectional SEM images (a’, b’, c’, d’) of the membranes, compared with those of the PVDF-g-PSSA membrane, the structures of the membranes with inorganic nanoparticles appears to be more compact and tighter. The reason for this may be due to the formation of a higher crosslinking density in the inorganic and organic network [47,48]. The granular structure and pores between the granules could also be clearly seen in the cross-sectional SEM images; perhaps they were the ion channels in the IEMs.

Furthermore, the surface of the membranes was flat and had no pinholes. Some white agglomerates appear on the surface of the membranes (a, b, c, d). This occurs because a small amount of undissolved PVDF or the excessive content of inorganic nanoparticles causes uneven dispersion during membrane formation. 

In short, a phase separation on the membrane surface was not observed in the SEM images, which indicated that the synthesized polymeric membranes were essentially homogeneous; that is, the nanoparticles blended into the membranes and did not form undesirable structures.

**Figure 6:**
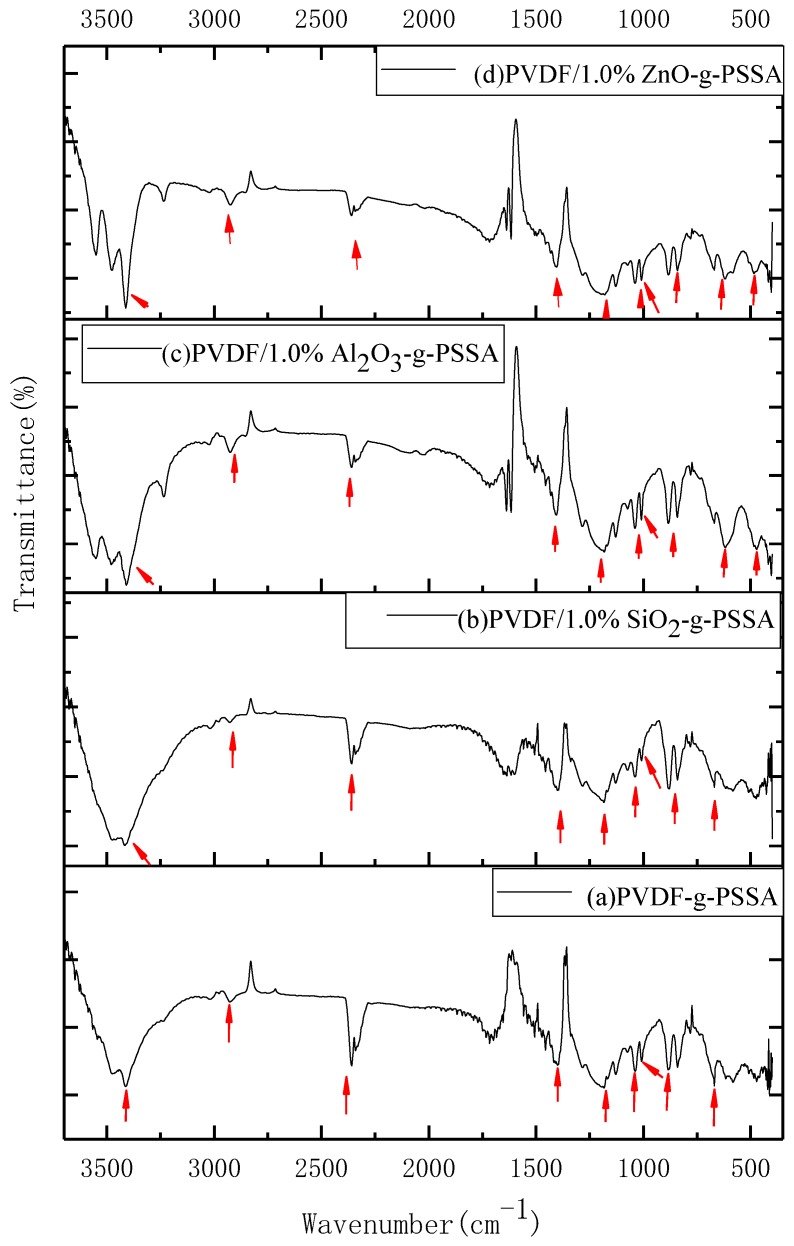
FTIR spectra of the prepared membranes (with the weight fraction 1.0% of inorganic nanoparticles taken as an example).

The FTIR spectra can verify the species of functional groups on the polymer chain in the prepared membranes. Figure 6 shows the spectra of PVDF-g-PSSA and PVDF/1% SiO_2_/Al_2_O_3_/ZnO-g-PSSA. As shown in Figure 6a, 1400 cm^−1^, 1180 cm^−1^, 1038 cm^−1^, and 884 cm^−1^ are the characteristic absorption peaks of PVDF. The band at 1506 cm^−1^ is the vibrational absorption peak of the benzene ring skeleton. The band at 669 cm^−1^ is the absorption peak for the replaced hydrogen on the benzene ring. The band at 2926 cm^−1^ is the hydrocarbon stretching vibration of methylene (–CH_2_–). The spectra show that styrene was grafted to the PVDF backbone. The band at 1007 cm^−1^ is the symmetrical stretching vibration of S–O in the –SO_3_ group, and the peak at 3446 cm^−1^ is attributed to the sulfonic acid group (–SO_3_), which can easily absorb water to form SO_3_H·H_2_O in air. The band at 2300 cm^−1^ is the absorption peak for –C≡C– which is minuscule in the alkalization process. The discussed groups and peaks (the red arrows in Figure 6a) are also shown in Figure 6b–d. Comparing these spectra indicates that the 1007 cm^−1^ band in Figure 6b (red arrow) might be attributed to Si–O–Si stretching. In Figure 6c, the 617 cm^−1^ and 470 cm^−1^ (red arrows) bands maybe caused by the Al–O stretching mode. As shown in Figure 6d (red arrow), the broad band at 485 cm^−1^ may be due to the Zn–O stretching mode.

### 3.5. Membrane Oxidative Stability and Burst Strength

**Figure 7:**
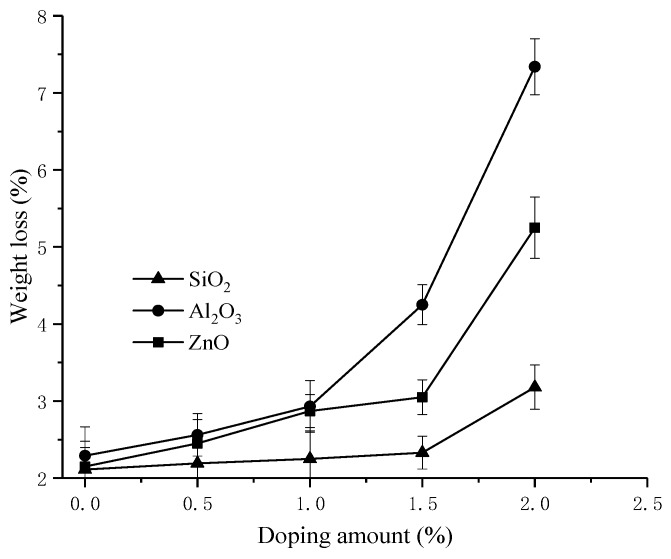
The weight loss values of the prepared membranes under different doping amounts of nano-SiO_2_, nano-Al_2_O_3_ and nano-ZnO.

**Figure 8:**
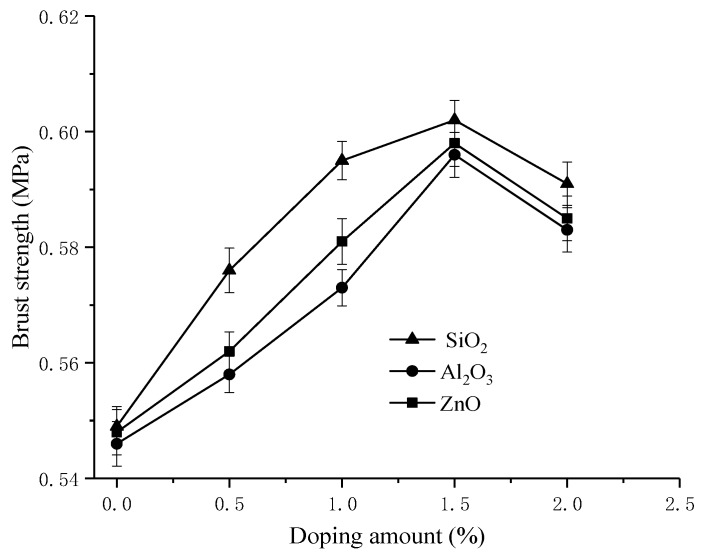
The effect to the burst strength curve of prepared membranes under different doping amounts of nano-SiO_2_, nano-Al_2_O_3_ and nano-ZnO.

The prepared membranes were immersed in an oxidant aqueous solution for the oxidative stability measurements. The oxidative stability of the membranes was characterized by their weight loss. A lower weight loss indicated a higher oxidative stability. The weight losses and the burst strength of the prepared membranes are shown in Figure 7 and Figure 8.

As shown in Figure 7, the PVDF-g-PSSA membrane had high oxidative stability (low weight loss). However, blending with SiO_2_/Al_2_O_3_/ZnO nanoparticles resulted in increased weight losses of the prepared membranes. This may be attributed to the adsorption characteristics of the blended membranes. The increased WU and IEC of the prepared membranes may lead to the strong possibility of oxidants diffusing into the membrane matrix, causing more weight loss in the prepared membranes. In contrast, the membranes blended with nano-SiO_2_ had the best antioxidant properties, which may be due to the better performance and strong chemical bonds of the prepared membranes. A similar result was found by Khodabakhshi et al.; that is, the minimum weight loss of membranes blending PVDF and SPPO was 23.5% [33]. The weight loss of PVDF 1.5% SiO_2_-g-PSSA in this paper was 2.33% and was only increased by 10% compared to the PVDF-g-PSSA membranes, signifying its better oxidation resistance.

Figure 8 reveals a slight change to the burst strength of the membranes. As the content of inorganic nanoparticles increased, the burst strength gradually rose until the nanoparticle additions reached 1.5%, and then began to decline. This may be because inorganic nanoparticles can improve the hydrophobicity of PVDF and make the membrane more flexible. However, excess inorganic nanoparticles make the membranes brittle, although they are still better than PVDF-g-PSSA membranes. The burst strength of PVDF/1.5% SiO_2_-g-PSSA membranes increased by 9.6% compared to PVDF-g-PSSA membranes. Figure 7 and Figure 8 show the small standard deviations of oxidative stability and burst strength, which illustrate the stabilized mechanical property of membranes.

## 4. Conclusions 

In this study, cation-exchange membranes based on PVDF were prepared with different contents of SiO_2_/Al_2_O_3_/ZnO nanoparticles using a blending method, and sulfonic acid groups were introduced to the prepared membrane by grafting and a sulfonation reaction. The IEC and hydrophilic nature of these cation-exchange membranes increased with increasing nano-SiO_2_/Al_2_O_3_/ZnO content, which was supported by the increased WU and porosity. The membrane conductivity increased when nano-SiO_2_/Al_2_O_3_/ZnO was added. The IEC increased by 43% and the membrane resistance decreased by 22% in PVDF/2% Al_2_O_3_-g-PSSA membranes compared to PVDF-g-PSSA membranes. The cation-exchange membranes, in the presence of nano-SiO_2_/Al_2_O_3_/ZnO, showed better permselectivity compared with that of the membranes without blended nanoparticles. In particular, the permselectivity of PVDF/2% SiO_2_-g-PSSA membranes, PVDF/2% Al_2_O_3_-g-PSSA membranes, and PVDF/2% ZnO-g-PSSA membranes were improved respectively by 7.2%, 6.3%, and 9.8%. In short, a proper amount of inorganic nanoparticles in the membranes can improve the hydrophilicity and the electrical conductivity of the PVDF membranes, and reduce energy consumption in practical applications. Similarly, the inorganic nanoparticles in membranes can reduce the maximum size of membrane pores, thereby increasing the permeability of the membranes.

The PVDF/2% Al_2_O_3_-g-PSSA membranes showed the best IEC of 2.735 mmol·g^−1^ and lowest membrane resistance of 7.1 Ω·cm^2^. They can be applied in wastewater treatment or electro-driven separation for the lowest possible energy consumption, among other applications. The PVDF/2% ZnO-g-PSSA membranes exhibited better permselectivity at 94.9%, and a transport number of 96.89%, thus, they have potential for applications in desalination and treatment of chloride-containing heavy metal wastewater to restrain the production of chlorine. The PVDF/1.5% SiO_2_-g-PSSA membranes showed a good oxidation resistance of 2.33% and other excellent properties. These membranes could be used in some alkali battery fields and electrochemical separation under harsh conditions, such as strong acid, strong alkali, and strong oxidation conditions.

## Figures and Tables

**Table 1:** Membrane potential and transport number values for different PVDF/X% SiO_2_/Al_2_O_3_/ZnO-g-PSSA membranes.

Membranes	Membrane Potential (mV)	Transport Number (%)
PVDF-g-PSSA	45.61	91.83
PVDF/0.5% SiO_2_-g-PSSA	46.20	93.66
PVDF/1.0% SiO_2_-g-PSSA	46.59	94.88
PVDF/1.5% SiO_2_-g-PSSA	46.75	95.36
PVDF/2.0% SiO_2_-g-PSSA	46.85	95.67
PVDF/0.5% Al_2_O_3_-g-PSSA	46.02	93.11
PVDF/1.0% Al_2_O_3_-g-PSSA	46.38	94.21
PVDF/1.5% Al_2_O_3_-g-PSSA	46.65	95.06
PVDF/2.0% Al_2_O_3_-g-PSSA	46.75	95.36
PVDF/0.5% ZnO-g-PSSA	46.30	93.96
PVDF/1.0% ZnO-g-PSSA	46.75	95.36
PVDF/1.5% ZnO-g-PSSA	47.18	96.71
PVDF/2.0% ZnO-g-PSSA	47.24	96.89
